# Mr. Dennett, on the Influenza

**Published:** 1803-04-01

**Authors:** C. Dennett

**Affiliations:** Soho Square


### *To the Editors of the Medical and Physical Journal.*

GENTLEMEN,

I Am fearful you will think I trespass too much on your Journal, in offering another Paper before the former, one you have done me the favour to acknowledge, has been published; but, on perusing the Number for the present month, I find an account of the epidemic raging at Paris; which to me appears similar to the complaint now general in this metropolis, (and called by some the Influenza) except that here both sexes are equally liable to it.

The treatment of practitioners I have found materially to differ, and the termination uncertain; but perceiving very early, that bleeding appeared improper, and having adopted a method attended with uniform success, is the only apology I can offer for sending you these observations; and whether my ideas are well founded or not, I hope the intention will appear to have arisen from an humble attempt to relieve the afflicted, as I do not presume to add any thing new.

The general symptoms I need not describe. After attending a few patients, I was satisfied in my own mind, that the complaint was chiefly confined to the membranous ous lining of the trachea, &c. therefore, my practice was to promote expectoration, using demulcent drinks, and avoiding every thing heating. In one case only, where the inflammatory peripneumonic symptoms appeared strong, I was induced to bleed; which was approved of by further advice called in the next day; and though the patient appeared relieved for a time, yet I was convinced it had done injury, as the expectoration was checked, and the cough and dyspnœa more troublesome, attended with great debility. The long illness of this man induced me to decline the lancet, and repeated observation in the cases where it has been ordered by superior authority, has confirmed my former opinion. Cupping is less objectionable, but even that, where much blood has been taken away, has to me appeared to have done mischief. Having said thus much, with diffidence, I beg to state my general practice.

Where the dyspnœa was great, a blister has been applied to the sternum, and in some cases repeated. The medicine given was ipecacuanha wine and tincture of squills, mixed with a solution of spermaceti or mucilage of gum arabic, in as large doses as the stomach would bear; and when the stomach has been very irritable, have added prepared kali with great advantage. For three weeks past I have attended a considerable number daily, afflicted with this complaint; and in no one instance has the above plan failed of quick relief, where the patient had not been bled.

In the first cases I employed the true James's powder, and also tartarized antimony, but found both affect the stomach and bowels too much, though given in the usual doses. I am, &c.

*Soho Square, March* 11, 1803. C. DENNETT.

